# Patulous eustachian tubes and an unusual case of fused retropharangeal internal carotid arteries with an aberrant course through the clivus and dorsum sellae

**DOI:** 10.1259/bjrcr.20190017

**Published:** 2020-02-12

**Authors:** Ignatious Tshegofatso IT Menyatsoe, Nausheen N Khan

**Affiliations:** 1 Diagnostic Radiology and Imaging, University of Pretoria, Hatfield, Pretoria, South Africa

## Abstract

Aberrant course of internal carotid arteries (ICA) is rarely seen. In patients who are asymptomatic, anomalies may be detected incidentally during head and neck examination. Symptomatic patients may present with hearing loss, pulsatile tinnitus, dysphagia or a foreign body sensation in the posterior pharynx. If the retropharyngeal course of ICA remains undiagnosed, accidental biopsy or surgical intervention can result in life threatening complications. The abnormal course of ICA results from a complex defect in embryological development and is unlikely to be an acquired process. Previously, bilateral and unilateral agenesis, hypoplastic, retropharyngeal tortuous ICA and kissing sellar ICA have been described in literature. We used various imaging techniques to describe this first case of fused ICA with an aberrant course through the clivus and dorsum sellae. The patient also presented with patulous Eustachian tubes on both the left and right side.

## Case report

A 32-year-old female patient presented with progressively deteriorating hearing loss, repeated ear infections and chronic cough since early childhood. The patient described worse cough in the morning, preceded by a feeling of irritation in the throat. The patient had no medical history or history of surgical intervention. The patient had bilateral hearing loss, confirmed with audiogram and tympanogram. Otoscopy confirmed bilateral dry tympanic membranes. The right tympanic membrane presented with complete myringosclerosis, while the left tympanic membrane was affected only on the anteroinferior aspect. The medical team requested further investigation using high resolution temporal bone CT. After assessing the CT findings, the medical team requested a computerized tomographic angiogram (CTA) and MRI.

## Imaging findings

**Figure 1.**
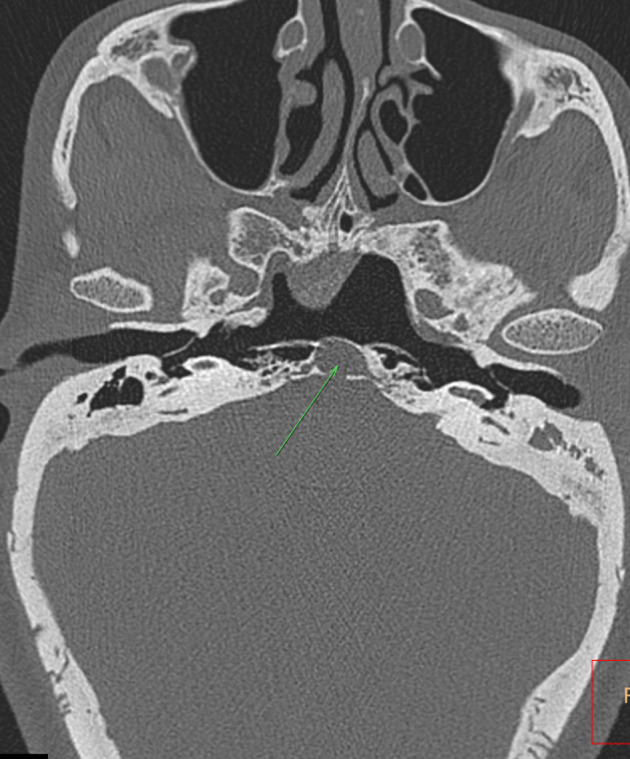
Transverse high resolution CT scan through the base of the temporal bone demonstrating large pharyngotympanic tubes with clear communication with the middle ear. Note absent carotid canals and a round defect in the body of the Clivus (arrow in Figure 1).

**Figure 2.**
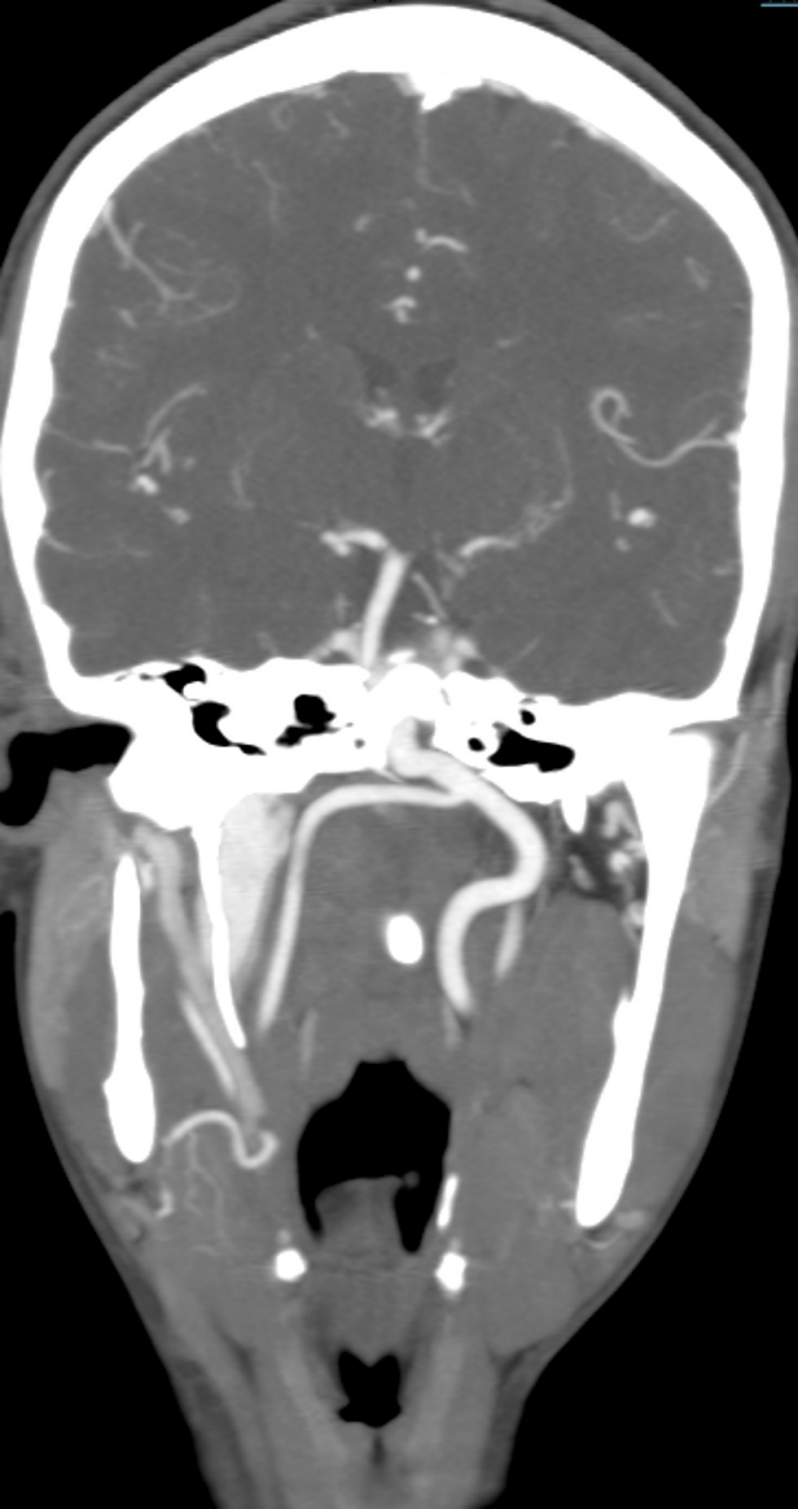
Coronal MIP CTA through the base of the skull shows the extracranial (ICA) to be tortuous, with right crossing to the left and fusing with the contralateral carotid in the retropharyngeal space. CTA, CT angiography; ICA, internal carotidartery; MIP, maximum intensity projection.

**Figure 3.**
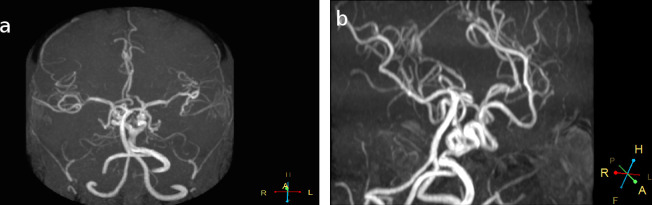
Coronal & sagital reformatted MIP MRI images demonstrationg the extracranial fusion of right and left ICA into a single vessel. It divides into very tortuous intracranial ICA’s. ICA, internal carotid artery; MIP, maximum intensityprojection.

CT scan demonstrated prominent bilateral pharyngotympanic tubes (Figure 1) with clear and open communication between the middle ear and nasopharynx. Normal Eustachian tubes were not seen. The ossicular chain and the contents of the inner ear were normal. CT scan revealed signs of chronic otomastoiditis accounting for the patient’s clinical presentation. The patient did not have normal carotid canals, and a round defect was noted on the body of the clivus (Figure 1), indicating the need for a CTA. The CTA revealed tortuous extra cranial internal carotid arteries (ICAs), with right ICA crossing to the left and fusing with the contralateral carotid artery in the retropharyngeal space (Figure 2), as also noted on reformatted maximum intensity projection MR angiography (MRA) images (Figure 3a & b).

**Figure 4.**
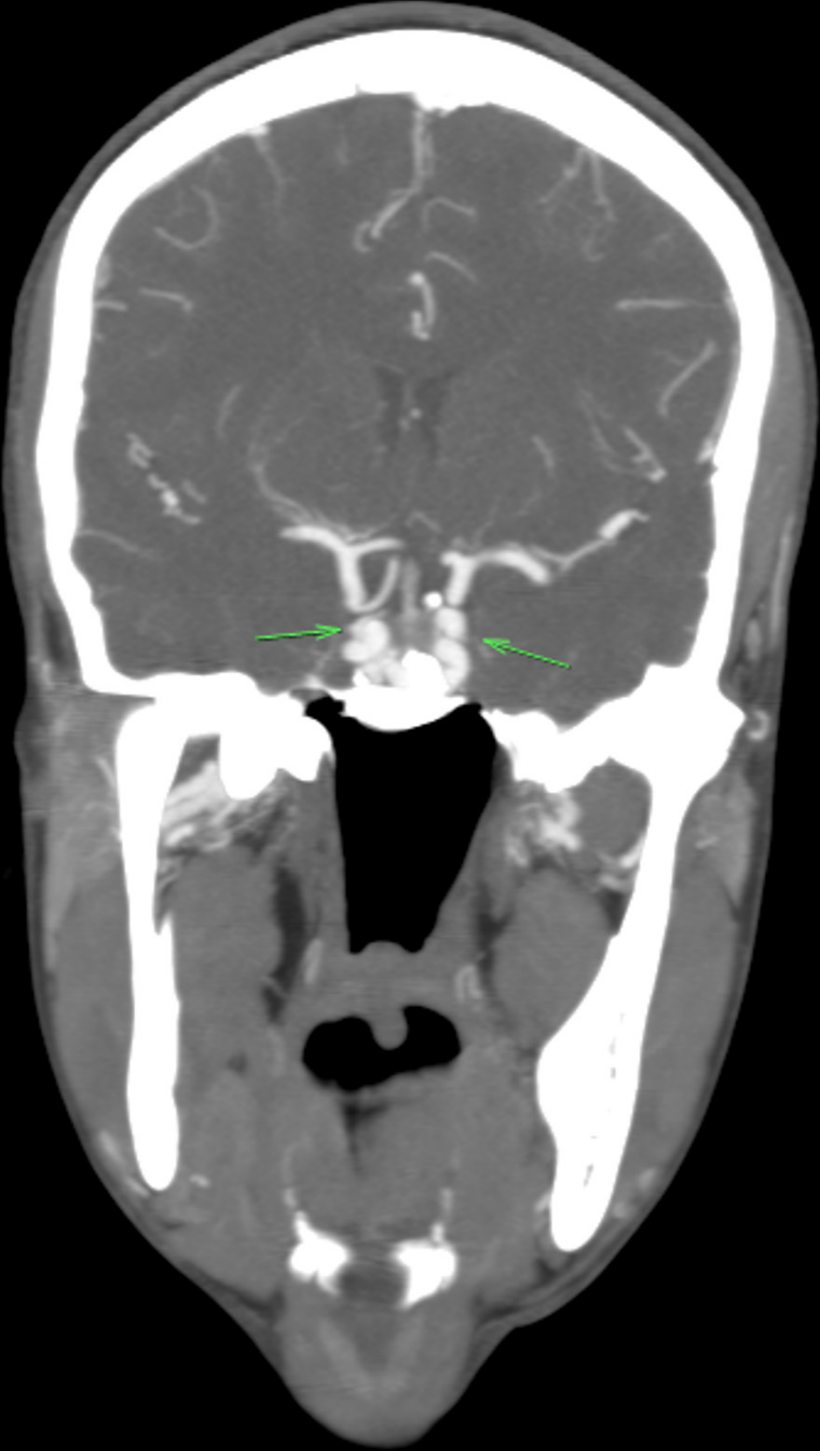
Coronal reformatted CTA image demonstrating a fused single ICA that runs through a solitary canal in the body of the clivus and bifurcates at dorsum sellae into right and left intra cranial very tortuous ICA’s as they continued intracranially to form respective arteries of the circle of Willis. CTA, CT angiography; ICA, internal carotidartery.

**Figure 5.**
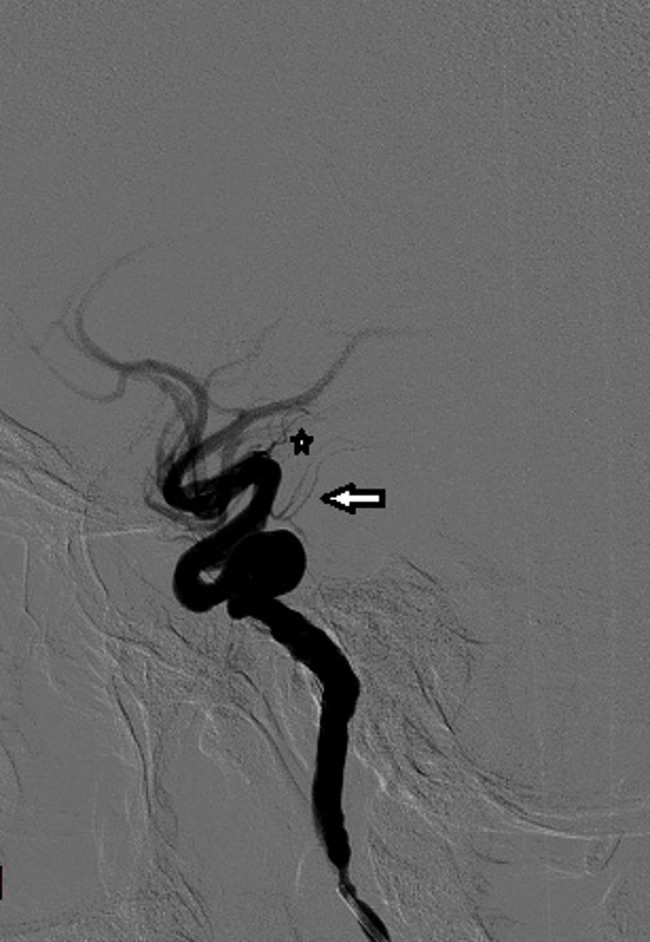
DSA lateral image showing small meningohypophyseal artery (arrow) and a normal pituitary blush (star). The ICA anastomosis is proximal to these vessels. DSA,digital subtraction angiogram; ICA, internal carotid artery.

The fused single ICA then ran through a solitary canal in the body of the clivus (Figure 1). The ICA bifurcated at the dorsum sellae into very tortuous right and left intra cranial ICAs (Figure 4), continuing intracranially to form the respective component arteries of the circle of Willis. The ICAs had no aneurysmal dilatations. Digital subtraction angiogram (DSA) was requested to exclude other congenital vascular abnormalities that may have contributed to the abnormality described above. The ascending pharyngeal arteries were normal with no sign of hypertrophy or aberrant course to suggest anastomotic abnormality. There were no signs of a clival branch of prominent neuromeningeal trunk continuing as a midline single ICA. The meningohypophyseal arteries on both sides were small (Figure 5). There was no sign of persistent hypoglossal artery and the hypoglossal canals were normal and not enlarged.

## Discussion

In this case, we describe a fused ICA with an aberrant course through the clivus and dorsum sellae. The described abnormalities may be due a complex defect in embryological development and are unlikely to be an acquired pathology.

**Figure 6.**
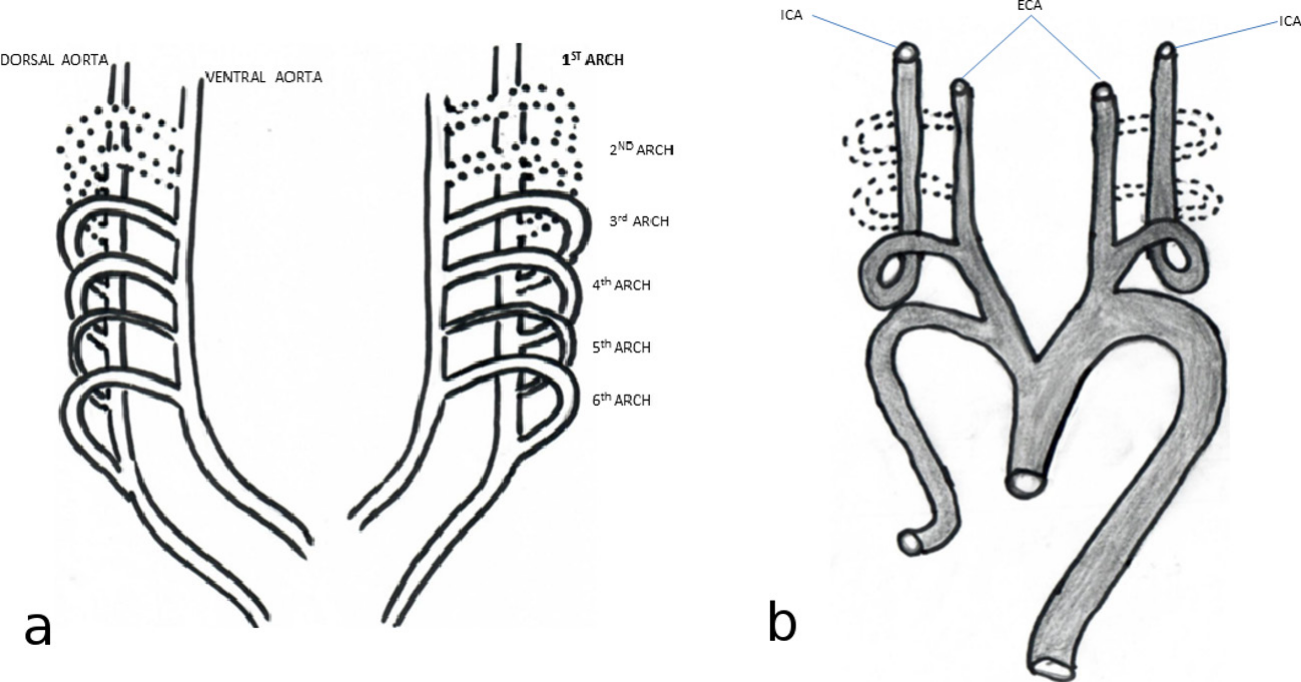
AorticNausheen N Khan, MBBS, FCRad Diag (SA) arches and dorsal aortae before transformation (a) into definite vascular system (b). Broken lines, represent the obliterated segments. The Vth arch forms incompletely. ICA,internal carotid artery.

In humans, embryonic development, occurring in the first 90 days following blastocyst development, constitutes a complex array of process including vascularisation and organ development. The ICAs usually present close to the 28th day of embryonic life. The process begins close to Day 20, when the growing embryo, unable to satisfy its nutritional demands, forms blood islands on either side of the embryonic shield, parallel and close to the embryonic midline. These islands later form a pair of longitudinal vessels known as dorsum aorta. During embryogenesis, the pharyngeal pouches form in a craniocaudal sequence, alongside the formation of the respective aortic arches. The aortic arches are embedded in the mesenchyme of the pharyngeal arches and terminate in the right and left dorsum aorta (Figure 6a). Aortic arches are connecting arteries between the ventral and dorsal arch, which go through complex vasculogenesis: annexation, regression and neovascularization around Day 20–28 of embryonic life.^1–3^

In the embryo, the third aortic arch gives rise to the common carotid arteries and the proximal ICA, while the cranial part of the dorsal aorta gives rise to the distal ICA. The external carotid arteries sprout from the common carotid arteries and receive contributions from the first and second aortic arches (Figure 6a and b). As the dorsal aorta descends into the chest, the course of the ICA straightens out. If the dorsal aorta does not descend completely, the ICA will follow a tortuous course.^1–4^ This may explain why the intra and extracranial ICA in our patient were tortuous.

Aside from tortuous ICAs, our patient presented with a fused ICA. During embryogenesis, the distal dorsal aortas usually fuses to form the abdominal aorta, while the cranial dorsal aorta forms the distal ICA. At no stage during normal embryogenesis, do the right and left cranial dorsal aorta fuse or share a common origin.^1^ We propose that the fusion of the ICA seen in our patient arose due to a defect in the embryonic plate causing the right ICA to cross-over to the left side. It is likely that the fusion of the ICAs preceded the development of bony tissue, resulting in absent carotid canals.

The lack of carotid canals in our patient supports the congenital origin of our observations. Embryologically, the base of the skull and foramina develop from the neurocranium or chordal chondrocranium portions. This occurs close to Day 28, which is around the same time as ICA formation. The presence of a developing ICAs is essential for the formation of carotid canals, and the canal is absent in cases of agenesis.^1,3,5–7^

Alternative to anastomosis of the ICAs, we may be seeing complete agenesis, or failed development, of both ICAs with canalization and enlargement of both ascending pharyngeal arteries which arise from the primitive vertebra-basilar system. Interestingly, anastomosis of the pharyngeal arteries and the ICA are common during embryologic development, with most of these anomalies resolving spontaneously.^8^ Failure to resolve may result in vascular anomalies and increased risk of aneurysm later in life. In our patient, the pharyngeal arteries could have fused in the retropharyngeal space, and formed its own transclival skull base canal, running via the clivus as a single artery. Persistent primitive anastomoses usually course via the foramen magnum or hypoglossal canal, and reports of transclival arteries are rare.^8^

Aside from vascular anomalies, our patient also presented with dilated and horizontally oriented Eustachian tubes. During embryonic development, the Eustachian tubes and tympanic cavity arises from the first pharyngeal pouch, at the same time as the carotids are developing, this pouch expands laterally where it comes into contact with the floor of the first pharyngeal cleft. The distal portion of the first pharyngeal pouch, or tubotympanic recess, widens and gives rise to a primitive tympanic cavity while the proximal part remains narrow to form Eustachian tubes. Any insult that occurs at this point can result in anomalies of both structures.^1^ Our patient presented with widening of the entire Eustachian tube on both sides with clear pharyngotympanic communication, possibly due to the first pharyngeal pouch failing to narrow medially thus resulting in abnormally widened Eustachian tubes. Absence of carotid canals and normal ICAs further creates a vacuum allowing the Eustachian tubes to dilate and occupy a more horizontal orientation.

Patulous pharyngotympanic tubes, or patulous Eustachian tubes, was first described by Jago in 1867 in patients with chronic illness including heart disease, diabetes, rheumatoid arthritis and cancer etc.^9,10^ Patulous Eustachian tubes are also seen in patients with Trisomy 13, 18, 21, 22; in patients with excessive weight loss, those on oral contraceptives and those with neurological disorders.^9,11^ Patulous Eustachian tubes have never been described as an anatomical abnormality due to embryological fault, which we speculate is the case in our patient. Abnormal patency of the Eustachian tube may cause excessive middle ear pressure changes, resulting in abnormal ossicular movements, leading to hearing impairment and may also expose the patient to episodes of repeated ear infections and chronic otomastoiditis as was the case in our patient.

Whilst uni- or bilateral agenesis, hypoplastic, retropharyngeal tortuous ICA, kissing sellar ICA have been described, we could not find any literature describing fused ICA with an aberrant course through clivus and dorsum sellae. The above-mentioned cervical and intracranial vascular anomalies and variants have been described extensively with satisfaction, including their clinical implications.^2–8,12–14^ We present two possible explanations for the observed vascular anomalies, but suggest that given the ascending pharyngeal arteries were normal with no sign of hypertrophy or aberrant course, our patients condition is more likely to be due to anastomosis of the ICA rather the pharyngeal arteries.

## Learning points

1. Aberrant course of ICAs although rare is seen with a large degree of variability. Often patients are asymptomatic and anomalies are detected incidentally on head and neck examination. The variation of ICA’s, if not recognized, may have disastrous life threatening implications, especially high risk for aneurysm. As radiologists we must be aware of such anomalies so as to alert treating physicians.
2. The presence of congenital variation of ICAs if noted on CT, MRI and DSA should prompt radiologists to search for other abnormalities that may develop at same stage of embryological development.
